# High Override Rate for Opioid Drug-allergy Interaction Alerts: Current Trends and Recommendations for Future

**Published:** 2015

**Authors:** Maxim Topaz, Diane L. Seger, Kenneth Lai, Paige G. Wickner, Foster Goss, Neil Dhopeshwarkar, Frank Chang, David W. Bates, Li Zhou

**Affiliations:** a Brigham and Women’s Hospital, Boston, MA, USA; b Harvard Medical School, Boston, MA, USA; c Clinical Informatics, Partners eCare, Partners Healthcare System, Boston, MA, USA; d Division of Rheumatology, Immunology and Allergy, Brigham and Women’s Hospital, Boston, MA, USA; e University of Colorado, Department of Emergency Medicine, Aurora, CO, USA

**Keywords:** Decision Support Systems, Clinical Drug Hypersensitivity/Prevention & Control, Drug Therapy, Computer-Assisted, Drug-Related Side Effects and Adverse Reactions, Hospital Information Systems, Medical Records Systems, Computerized, Reminder Systems

## Abstract

This study examined trends in drug-allergy interaction (DAI) alert overrides for opioid medications - the most commonly triggered alerts in the computerized provider order entry (CPOE). We conducted an observational analysis of the DAI opioid alerts triggered over the last decade (2004–2013, n=342,338) in two large academic hospitals in Boston (United States). We found an increasing rate of DAI alert overrides culminating in 89.7% in 2013. Allergic reactions included a high proportion (38.2%) of non-immune mediated opioid reactions (e.g. gastrointestinal upset). The DAI alert override rate was high for immune mediated (88.6%) and life threatening reactions (87.8%). Exact allergy-medication matches were overridden less frequently (about 70%) compared to non-exact matches within allergy groups (over 90%). About one-third of the alert override reasons pointed to irrelevant alerts (i.e. “Patient has tolerated the medication before”) and 44.9% were unknown. Those findings warrant further investigation into providers’ reasons for high override rate. User interfaces should evolve to enable less interruptive and more accurate alerts to decrease alert fatigue.

## Introduction

During the past several decades, computerized provider order entry (CPOE) systems have become a standard and are now required for each medication order in many countries (e.g. Meaningful Use requirement in the US) [1]. These systems are vital in providing better quality care and reducing medical errors [2]. One of the important features of CPOE systems is to help providers avoid prescribing medications their patients are allergic to. This is done through drug-allergy interaction (DAI) alerts in the electronic health records. However, constant interruptive DAI alerts might overwhelm providers and cause alert fatigue - a common phenomenon where clinicians become desensitized and tend to delay or miss the response to the alarms [3,4]. There is only limited evidence on the issues related to design and implementation of DAI alerts. This study examines the rates and reasons for DAI alert overrides for one of the most common types of alerts - opioid medications.

## Background

Previous evidence suggests that DAI alerts involving opioid analgesics - or narcotics - are among the most common types of alerts encountered by health practitioners [5–7]. However, true immune mediated reactions to opioids are rare and most of the opioid related reactions are non-immune mediated sensitivities or opioid induced adverse events [8,9]. True immune mediated reactions to opioids are often IgE mediated or T-cell mediated and include hives, rash, severe hypotension, bronchospasm, angioedema, and anaphylaxis [8,9]. Non-immune mediated or mimic immune reactions to opioids are usually caused by endogenous histamine release from the mast cells and cause urticaria, flushing, sweating, vasodilation, and bronchoconstriction [8,9]. Such reactions to opioids are often idiosyncratic; they may or may not recur when patients take the same opioid medication again. Other non-immune mediated side effects include nausea/vomiting, constipation, sedation, delirium, respiratory depression, and urinary retention [8,9]. In CPOE systems, a significant proportion of opioid DAI alerts are not generated by an exact match between the allergy and prescribed medication (e.g. Codeine→Codeine), but rather represent a possible cross-reactivity association between medications in similar drug families (e.g. Codeine→Oxycodone). For these reasons, opioid DAI alerts are often non-specific and might potentially increase “alert fatigue” among providers.

In the past, studies have shown that DAI alerts are frequently overridden by providers [10]. For example, two previous studies from Brigham and Women’s Hospital in Boston have identified an increasing DAI alerts override trend since introduction of CPOE system over two decades ago. The overall override rates increased from about 50% in 1995 [5] to about 80% in 2002 [6]. Also, studies have shown that providers are less likely to override allergic reactions generated by an exact prescribed medication-allergy match [6, 7]. In this paper, we aimed to follow-up on this previous work and provide an update on the trends in opioid DAI alert overrides in the past 10 years. Our goals were to: 1) describe prevalence rate of opioid DAI alert overrides 2) examine the nature of opioid-related allergic reactions (immune mediated vs. non-immune mediated; potentially life threatening vs. non-life threatening) and 3) examine factors associated with providers’ tendency to override opioid DAI alerts (exact drug-allergy match vs. cross reactivity vs. drug class).

## Methods

For this observational study we used data from two large academic medical centers (Brigham and Women’s Hospital and Massachusetts General Hospital) from Partners Healthcare, an integrated healthcare system in the Boston area. The data generated during 10 years (2004–2013) was extracted from Partners Enterprise-wide Allergy Repository (PEAR), a longitudinal allergy database shared within the Partners provider/hospital network [11].

In PEAR, medications are encoded using a combination of local (Partners Master Drug Dictionary) and proprietary (First Databank, Inc.™) terminologies. For each patient admitted, providers are required to enter allergy information or indicate no known allergies. The recorded information combines both patient and provider reported reactions. Allergic reactions are also entered as structured or unstructured (free text) information for each allergen. Every prescribed medication is automatically checked against possible allergies in PEAR.

Drug-allergy interaction alerts are triggered when a prescribed medication matches stored allergy information in either: 1) an exact manner (definite match between main medication ingredient and allergen, i.e. Codeine→Codeine) or 2) a probable manner (probable match where allergen matches allergy group of one or more of the prescribed medication ingredients, i.e. Codeine→Oxycodone). DAI alerts present the provider with several information components, including: the allergy; the allergen; nature of the allergy-allergen match (definite/probable); and patient’s reaction(s), if known. When presented with the drug-allergy interaction alert, provider needs to decide whether to cancel the medication or to override the alert. Several structured dropdown options (i.e. “Patient has tolerated drug previously”) are available for the override reason together with a free text option. Providers decisions and alert information are then stored in PEAR for future reference.

Some allergy-reaction combinations are overridden less often than others (i.e. allergies resulting in anaphylaxis vs. nausea). To account for this, our research team has classified allergic reaction information into two categories based on the literature review [8,9] and consultations with allergy specialists: 1) likely immune mediated (i.e. rash or anaphylaxis) or non-immune mediated (i.e. nausea or vomiting) and 2) potentially life threatening (i.e. anaphylaxis or bronchospasm) or non-life threatening (i.e. gastrointestinal upset or nausea).

All data including allergies, allergens, medication groups, and DAI alert override rates/reasons was kept and managed using Microsoft SQL Server Management Studio [12]. For aim 1 of this study, we queried the PEAR database and summarized the frequencies of opioid DAI alerts by year. Aim 2 was achieved by summarizing the frequencies of life threatening and immune-mediated allergic reactions. For aim 3, we first queried PEAR database for medication-allergen match type (definite/probable). We then compared the frequencies of DAI alert overrides for immune-mediated and life threatening reactions by allergy-medication match type. Statistical procedures included t-tests and chi-square tests, when appropriate. The statistical analysis and group comparisons were conducted in STATA v.11 [13]. Lastly, we summarized the frequencies of the DAI alerts override reasons recorded by the providers.

This study was approved by Partners Institutional Review Board (IRB).

## Results

### Aim 1: Describing the prevalence and rates of opioid DAI alert overrides

**Figure 1 –:**
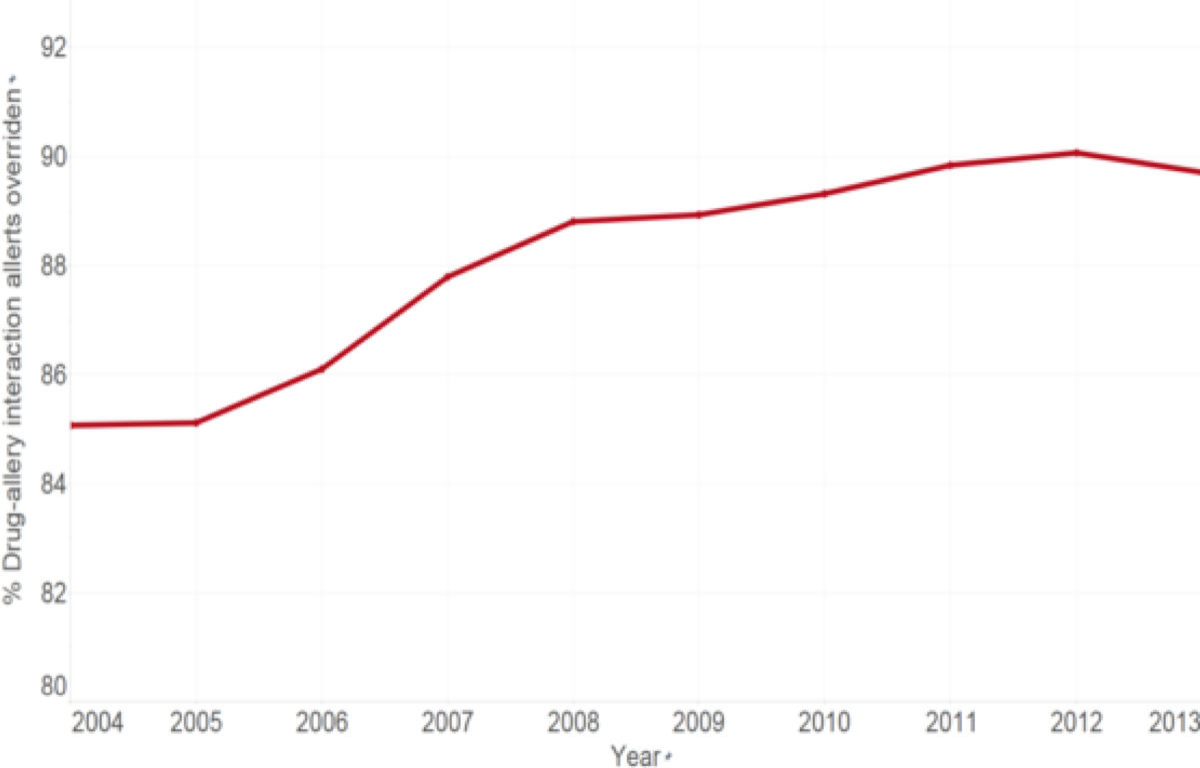
Percentage of drug-allergy alert overrides for opioids over the last decade

Overall, the PEAR database included about one million drug-allergy interaction alerts between 2004–2013. Of these alerts, 37.3% (n=355,179) were generated for the allergens that belong to “Analgesic Narcotic Agonists” drug class (e.g. Codeine, Oxycodone, Morphine, Hydromorphone, Tramadol). This was the most common class of drugs generating drug-allergy interaction alerts in our database. While the general override rate was 83.9% for all medications, opioid DAI alerts were overridden in 88.8% cases. Figure 1 presents the increasing proportion of overrides over the years, from 85.1% in 2003 to 89.7% in 2013. We also identified a constant increase in the overall number of opioid DAI alerts from 10,500 in 2004 to 55,048 in 2013.

### Aim 2: Examining the nature of opioid-related allergic reactions

Table 1 presents the frequencies of the 15 most common opioid allergy reactions (covering 98% of all the reactions). Each reaction is also classified into likely immune mediated and potentially life threatening categories. 40.6% of the reactions were likely immune mediated (38.2% non-immune mediated, and the rest 21.1% unknown or not available) while only 10.4% were potentially life threatening.

### Aim 3: Examining factors associated with provider’s tendency to override opioid drug-allergy interaction alerts

First, we examined whether there was a difference in override rates for previously reported immune mediated and life threatening reactions versus non-immune mediated and non-life threatening reactions. Although the DAI alerts override rates were statistically different, the clinical difference was only marginal for either immune mediated reactions (immune mediated overrides 88.6% vs. non-immune mediated overrides 89%, p<.001) and for the life threatening reactions (life threatening 87.8% overrides vs. non-life threatening 89% overrides, p<.001).

Further investigation revealed that majority of DAI alerts were triggered by the probable match medications (allergen matches the allergy group of one or more of the prescribed medication ingredients, 87% of DAI alerts) rather than definite match. To account for this, we conducted further comparisons based on allergy-medication match status.

We found that providers were significantly more likely to override probable matches for both immune mediated (90.7%) and non-immune mediated (91.3%) reactions whereas definite matches were overridden less frequently (72.4% immune mediated, 75.8% non-immune mediated). Similar differences (Table 2) were discovered based on life threatening reaction status which were the least overridden combination (69.9% overrides). All comparisons were statistically significant at p<.001.

In 44.9% of DAI alert overrides, we found no override reason entered by the practitioner (Table 3). Among the provided override reasons, 29% indicated previous tolerance or no allergy to the prescribed medication and 7.1% indicated that there was no reasonable alternative (provider will monitor for reaction). The remaining 17.2% of the reasons were presented as other free text with entries such as “OK with patient” or “Pain protocol”. Since one-third of the cases were indicated as either previous tolerance or no existing allergy, we compared the rates of immune mediated and life threatening reactions override without those two override groups. Override trends (not shown) were similar to those presented in Table 2.

## Discussion

Our findings confirm a concerning trend of continuously increasing drug allergy alert overrides over the past few decades. Since the introduction of DAI alerts as a core feature of CPOE, the override rates increased from about 50% in 1995 [5], to about 80% in 2002 [6], culminating in the current rate of about 90% in 2013 in our study. We also identified an overall four-fold increase in the number of opioid DAI alerts over time. To our best knowledge, no major changes were made to the CPOE system during the study time period. This finding is consistent with the recent studies reporting a constant increase in the opioid drug prescription rates over time in inpatient and outpatient settings. For example, several recent studies have reported that opioid prescriptions increased as high as three-fold in the past two decades in the US [14–17]. It is possible that the increase in the DAI override rates is associated with an increase of opioid prescriptions. Also, opioid DAI alerts are the most common type of alerts, and providers might override more of them due to alert fatigue – a well-known phenomenon where providers become overwhelmed with the constant interruptive alerts and override more of them over time [3,4,10].

Further investigation into the nature of the opioid-related reactions has also reveled several further concerning trends. First, only about 40% of the indicated reactions were likely a result of immune-mediated processes (i.e. IgE mediated or T-cell mediated reactions such as anaphylaxis or rash). Another 40% of the reactions were likely opioid side effects, such as gastrointestinal upset or nausea and the remaining reactions were unknown or unavailable. In our study, both types of reactions were overridden in approximately 89% of the cases. Presenting accurate information has the potential to increase users buy-in to the system [18,19]. However, in our case, an inaccurate mix of allergy and side effects-related reaction information may have increased the rate of DAI alert overrides. In addition, providers might need to pay more attention to capturing reliable allergic reaction information to avoid further inconsistencies. Potentially, clinical decision support tools might help in identifying and presenting the non-immune mediated reactions differently, either at the point of allergic reaction entry by the provider or when the DAI alert is presented [19].

In an attempt to better understand providers’ decision making when overriding DAI alerts, we also grouped reactions into potentially life threatening reactions versus non-life threatening reactions. Although only one-tenth of the reactions were potentially life threatening, we were surprised by the high rate of overrides. Here again, the high DAI override rates for life threatening and non-life threatening reactions were almost identical. This again underlines a critical need in decreasing the alert fatigue [4,7] and further exploration of users’ experiences.

Another important aspect of our study was to examine the override rates by allergen-prescribed medication match. Similar to other reports [6], only about one-tenth of alerts were triggered by an exact match while the rest were triggered by probable matches of medication-allergy group. Not surprisingly, providers were significantly more likely to override probable matches, especially for non-immune mediated and non-life threatening reactions. Definite match allergy-medication combinations, especially those with life threatening reactions, were overridden the least, in about 70% of the cases. Still, the override rate was notably high and there is a need for further investigation, potentially using qualitative methods to better understand providers’ reasons for overriding even the most alarming DAI alerts.

Finally, examining providers’ reasons to override DAI alerts, we found that about one-third of the alerts were triggered for medications that patients have either previously tolerated or had no allergy to. Clearly, CPOE user interfaces must evolve to learn or negate medications patients have tolerated or had no allergy to in the past so providers are not inundated with alerts to previously tolerated medications [2]. Of note, we did not identify changes in DAI alert override patterns when we repeated the analysis without those categories. Another concerning trend was the lack of override reasons for about 45% of alerts. This finding raises several liability and ethical questions and warrants careful re-examination of the user interface to improve the capture and storage of DAI alert override reasons. Training of providers on importance of information captured by electronic health records might be one of the keys to this issue [20,21]. Finally, we also conducted some preliminary interviews with the providers to better understand why alerts are frequently overridden. This anecdotal evidence suggests that inpatient providers are reluctant to remove or otherwise change patients’ allergies as they believe that this is a primary care provider’s responsibility.

## Limitations

This study is not without limitations. First, the analysis was limited to two large academic hospitals in Boston. In addition, different CPOE systems have different approaches to DAI alerting and use different medication vocabularies for drug-allergy interaction. Those factors might decrease the generalizability of our findings. Finally, our classification of immune mediated and life threatening opioid reaction is an estimation based on the captured patient reactions rather than a conclusive statement based on allergy tests.

## Conclusions

This study examined trends in drug allergy interaction alerts for the most common trigger – opioid medications. Over the past decade, we found an increasing rate of DAI alert overrides culminating in a 90% rate. Allergic reaction information in our database included a high proportion (over 40%) of non-immune opioid reactions (e.g. gastrointestinal upset). The DAI override rate did not seem to differ significantly for immune mediated and life threatening reactions. The only factor contributing to the decrease in overrides was the nature of the DAI alert: definite allergy-medication matches were overridden less frequently compared to probable matches. Finally, about one-third of the alert override reasons pointed to the irrelevant alerts (i.e. “Patient has tolerated the medication before”) and a significant proportion of DAI overrides were missing the override reason. Those findings warrant further investigation into providers’ reasons for overriding alerts. It is also clear that user interfaces and drug-alerting algorithms should evolve to enable less interruptive and more accurate alerts to decrease alert fatigue.

## Figures and Tables

**Table 1 –:** Opioid allergy reactions

Reaction	Freq.*	%	Likely immunemediated	Potentially life threatening
Hives or Rash	75,528	20.8	Yes	No
Gastrointestinal Upset	63,583	17.5	No	No
Itching	32,291	8.9	No	No
Mental Status Change	22,669	6.2	No	No
Vomiting	22,555	6.2	No	No
Nausea	21,820	6.0	No	No
Anaphylaxis	15,953	4.4	Yes	Yes
Shortness of Breath	5,175	1.4	Yes	Yes
Swelling (unspecified location)	4,725	1.3	Yes	No
Headaches	4,342	1.2	No	No
Angioedema	3,269	0.9	Yes	Yes
Hypotension	2,785	0.8	Yes	No
Bronchospasm or Wheezing	2,428	0.7	Yes	Yes
Seizures	1,829	0.5	No	Yes
Unknown/ unavailable	76,861	21.1	n/a	n/a
**Total**	**363,557**		**147,780**	**37,678**

* The total frequency of allergic reactions is larger than unique DAI alerts triggered since each unique DAI alert could have more than one associated reactions.

**Table 2 –:** Drug-allergy interaction overrides by definite and probable allergen-medication matches

Reaction type	Allergy/ Medicati on match	Medication canceled N(%)	Alert Overridden N(%)	Overall N(%)
**Immune mediated reactions to opioids**				
Likely NON-immune mediated	Definite	5454 (242)	17103 (75.8)	22557 (6.4)
	Probable	11442 (8.7)	120290 (913)	131732 (37.1)
Likely immune mediated	Definite	4048 (27,6)	10634 (72.4)	14682 (4.1)
	Probable	10251 (93)	100196 (90.7)	110447 (31.1)
Unknown	Definite	2298 (261)	6514 (73.9)	8812 (2.5)
	Probable	6349 (9.5)	60600 (90.5)	66949 (18,8)
**Life threatening reactions to opioids**				
Likely NON-life threatening	Definite	8301 (25)	24945 (75)	33246 (9.4)
	Probable	18335 (8.8)	1S9961 (912)	208296 (58.6)
Likely life threatening	Definite	1194 (30.1)	2775 (69.9)	3969 (1.1)
	Probable	3317 (10)	29802 (90)	33119 (93)
Unknown	Definite	2305 (26.1)	6531 (73.9)	8836 (23)
	Probable	6390 (9.4)	61323 (90.6)	67713 (19.1)
Total		39842 (112)	315337 (88.8)	355179

**Table 3 –:** Drug-allergy interaction alerts override reasons

Override reason	Freq.	%
Patient has tolerated drug previously	91,548	29.0
Patient reports no allergy	5,666	1.8
No reasonable alternative – will monitor for reaction	22,496	7.1
Unknown	141,437	44.9
Other (allows user to enter free text)	54,190	17.2
Overall	331,669	

## References

[1] Office of National Coordinator for Health IT. Meaningful Use: Computerized Physician Order Entry (CPOE) for Medication Orders. 2104; Available at: http://www.healthit.gov/providers-professionals/achieve-meaningful-use/core-measures/cpoe-meaningful-use.

[2] Kuperman GJ, Bobb A, Payne TH, Avery AJ, Gandhi TK, Burns G, Classen DC, Bates DW. Medication-related clinical decision support in computerized provider order entry systems: a review. J Am Med Inform Assoc 2007; 14(1): 29–40. 17068355 10.1197/jamia.M2170 PMC2215064

[3] Nanji KC, Slight SP, Seger DL, Cho I, Fiskio JM, Redden LM, Volk LA, Bates DW. Overrides of medication-related clinical decision support alerts in outpatients. J Am Med Inform Assoc 2014; 21(3): 487–491. 24166725 10.1136/amiajnl-2013-001813 PMC3994856

[4] Smithburger PL, Buckley MS, Bejian S, Burenheide K, Kane-Gill SL. A critical evaluation of clinical decision support for the detection of drug-drug interactions. Expert Opin Drug Saf 2011; 10(6): 871–882. 21542665 10.1517/14740338.2011.583916

[5] Abookire SA, Teich JM, Sandige H, Paterno MD, Martin MT, Kuperman GJ, Bates DW. Improving allergy alerting in a computerized physician order entry system. Proc AMIA Symp 2000: 2–6. 11080034 PMC2243998

[6] Hsieh TC, Kuperman GJ, Jaggi T, Hojnowski-Diaz P, Fiskio J, Williams DH, Bates DW, Gandhi TK. Characteristics and consequences of drug allergy alert overrides in a computerized physician order entry system. J Am Med Inform Assoc 2004; 11(6): 482–491. 15298998 10.1197/jamia.M1556 PMC524628

[7] Ariosto D Factors Contributing to CPOE Opiate Allergy Alert Overrides. AMIA Annu Symp Proc 2014: 256–265. 25954327 PMC4419937

[8] Joint Task Force on Practice Parameters- American Academy of Allergy, Asthma and Immunology. Drug allergy: an updated practice parameter. Ann Allergy Asthma Immunol 2010; 105(4): 259–273. 20934625 10.1016/j.anai.2010.08.002

[9] DeDea L Prescribing opioids safely in patients with an opiate allergy. JAAPA 2012; 25(1): 17. 10.1097/01720610-201201000-00003 22384750

[10] van der Sijs H, Aarts J, Vulto A, Berg M. Overriding of drug safety alerts in computerized physician order entry. J Am Med Inform Assoc 2006; 13(2): 138–147. 16357358 10.1197/jamia.M1809 PMC1447540

[11] Kuperman GJ, Marston E, Paterno M, Rogala J, Plaks N, Hanson C, Blumenfeld B, Bates DW. Creating an enterprise-wide allergy repository at Partners HealthCare System. AMIA Annu Symp Proc 2003: 376–380. 14728198 PMC1480073

[12] Microsoft™. Microsoft SQL Server Management Studio. 2008.

[13] StataCorp. STATA. v. 2010.

[14] Daubresse M, Chang HY, Yu Y, Viswanathan S, Shah ND, Stafford RS, Kruszewski SP, Alexander GC. Ambulatory diagnosis and treatment of nonmalignant pain in the United States, 2000–2010. Med Care 2013; 51(10): 870–878. 24025657 10.1097/MLR.0b013e3182a95d86 PMC3845222

[15] Mazer-Amirshahi M, Mullins PM, Rasooly I, van den Anker J, Pines JM. Rising opioid prescribing in adult U.S. emergency department visits: 2001–2010. Acad Emerg Med 2014; 21(3): 236–243. 24628748 10.1111/acem.12328

[16] Jena AB, Goldman D, Weaver L, Karaca-Mandic P. Opioid prescribing by multiple providers in Medicare: retrospective observational study of insurance claims. BMJ 2014;348:g1393. 24553363 10.1136/bmj.g1393 PMC3928962

[17] Manchikanti L, Helm S, Fellows B, Janata JW, Pampati V, Grider JS, Boswell MV. Opioid epidemic in the United States. Pain Physician 2012; 15(3 Suppl): ES9–38. 22786464

[18] Khajouei R, Jaspers MW. The impact of CPOE medication systems’ design aspects on usability, workflow and medication orders: a systematic review. Methods Inf Med 2010; 49(1): 3–19. 19582333 10.3414/ME0630

[19] Kuperman GJ, Gandhi TK, Bates DW. Effective drug-allergy checking: methodological and operational issues. J Biomed Inform 2003; 36(1–2): 70–79. 14552848 10.1016/s1532-0464(03)00063-7

[20] Campbell EM, Sittig DF, Ash JS, Guappone KP, Dykstra RH. Types of unintended consequences related to computerized provider order entry. J Am Med Inform Assoc 2006; 13(5): 547–556. 16799128 10.1197/jamia.M2042 PMC1561794

[21] Agency for Healthcare Research and Quality. Computerized Provider Order Entry - Inpatient Best Practices. 2013: Available at: http://healthit.ahrq.gov/ahrq-funded-projects/emerging-lessons/computerized-provider-order-entry-inpatient.

